# Increasing tree cover and high-albedo surfaces reduces heat-related ER visits in Los Angeles, CA

**DOI:** 10.1007/s00484-024-02688-4

**Published:** 2024-04-29

**Authors:** Scott Sheridan, Edith B. de Guzman, David P. Eisenman, David J. Sailor, Jonathan Parfrey, Laurence S. Kalkstein

**Affiliations:** 1 Kent State University, Kent, OH USA; 2 Division of Agriculture & Natural Resources and UCLA Luskin Center for Innovation, University of California, Los Angeles, CA USA; 3 David Geffen School of Medicine at UCLA and UCLA Fielding School of Public Health, Los Angeles, CA USA; 4 School of Geographical Science and Urban Planning, Arizona State University, Tempe, AZ USA; 5 Climate Resolve, Los Angeles, CA USA; 6 Applied Climatologists, Inc., Marco Island, FL USA

**Keywords:** Extreme heat, Heat-related illness, Urban heat island, Urban greening, Urban cooling, Heat mitigation

## Abstract

There is an urgent need for strategies to reduce the negative impacts of a warming climate on human health. Cooling urban neighborhoods by planting trees and vegetation and increasing albedo of roofs, pavements, and walls can mitigate urban heat. We used synoptic climatology to examine how different tree cover and albedo scenarios would affect heat-related morbidity in Los Angeles, CA, USA, as measured by emergency room (ER) visits. We classified daily meteorological data for historical summer heat events into discrete air mass types. We analyzed those classifications against historical ER visit data to determine both heat-related and excess morbidity. We used the Weather Research and Forecasting model to examine the impacts of varied tree cover and albedo scenarios on meteorological outcomes and used these results with standardized morbidity data algorithms to estimate potential reductions in ER visits. We tested three urban modification scenarios of low, medium, and high increases of tree cover and albedo and compared these against baseline conditions. We found that avoiding 25% to 50% of ER visits during heat events would be a common outcome if the urban environment had more tree cover and higher albedo, with the greatest benefits occurring under heat events that are moderate and those that are particularly hot and dry. We conducted these analyses at the county level and compared results to a heat-vulnerable, working-class Los Angeles community with a high concentration of people of color, and found that reductions in the rate of ER visits would be even greater at the community level compared to the county.

## Introduction

The Los Angeles (LA) region is experiencing an array of challenges caused or intensified by climate change. Of the many changes that are presently occurring or are anticipated in the near future, extreme heat threatens the largest number of people because many of the region’s residents lack the necessary resources to cope (Li et al. 2020; Chakraborty et al. 2019), which in turn raises the likelihood of experiencing heat-related health problems (Ostro et al. 2011). Continued warming is projected to increase average temperatures some 2–3 °C by mid-century, and by 3–5 °C by the end of the century, with temperature extremes manifesting both in the rising number of extremely hot days, and in the hottest days being up to 5 °C hotter than presently experienced (Hall et al. 2018). In LA’s climatically and topographically variable region, some neighborhoods will experience a sixfold increase in the number of extreme heat days (Hall et al. 2018).

During hot weather conditions, LA already sees all-cause mortality increase by 8%, in large part because heat places increased stress in individuals who suffer from a variety of underlying health conditions (Kalkstein et al. 2014). Back-to-back extreme heat days can have even more dangerous results, with all-cause mortality periodically increasing by 30% during and immediately following back-to-back days of extreme heat (Kalkstein et al. 2014). Extreme heat events that are longer-lasting and more severe are expected to occur more frequently in the future, creating conditions for potentially devastating health outcomes (Sheridan et al. 2012). The lack of nightly relief from the heat — a consequence of heat-retaining urban development — further exacerbates health risk as individuals exposed to elevated daytime temperatures are unable to get respite when the body seeks to rest and recover (Dousset et al. 2011).

Like many shifts brought on or augmented by climate change, in the United States heat raises equity concerns because it disproportionately impacts low-income urban communities and people of color (Jesdale et al. 2013). Neighborhoods with denser development tend to have older housing stock, low tree cover, and households with a lack of air conditioning or the inability to pay for it. These conditions further exacerbate the urban heat-island and contribute to a feedback loop of increased, sustained warming. This affects some communities more than others: respectively, Latino/a and Black communities are 21% and 52% more likely to be at risk of heat-related health problems, due largely to characteristics of the built environment (Jesdale et al. 2013). In the United States, the twentieth-century practice of redlining was a race-based evaluation of neighborhoods that resulted in otherwise creditworthy applicants being denied home loans (Federal Reserve Board 2006). Formerly redlined communities enjoy less tree cover and experience higher neighborhood temperatures compared to their non-redlined counterparts as the legacy of policies continues to shape outcomes decades later (Locke et al. 2021). Today, formerly redlined neighborhoods experience average temperatures that are hotter by approximately 2.6 °C (and up to 7.0 °C) compared with better-rated neighborhoods in the same city (Hoffman et al. 2020). In Los Angeles, inequity of heat impacts puts Black and Latino/a communities at significantly higher risk of heat-related illness and death as the climate warms (Kalkstein et al. 2014). During multi-day heat events, risk of heat-related death increases by 46% in Latino/a communities and 48% in elderly Black communities, compared with an increase of 22–26% in communities that are not Latino/a or Black (Kalkstein et al. 2014).

While the risks of extreme heat continue to grow in a warming climate, effective cooling strategies to alleviate urban heat exist and are within reach. For example, institutional risk mitigation strategies such as heat alerts and warnings can improve health outcomes during extreme heat events, and built environment interventions can reduce urban temperatures through increased vegetation, improved building standards, and increased reflectance of the buildings, roads, and other surfaces (Keith et al. 2020).

Trees mitigate heat through the mechanisms of shading, absorbing reflected light heat from surrounding surfaces, and transpiring heat (O’Malley et al. 2015). Tree shade intercepts direct shortwave radiation and prevents it from heating surfaces, reducing surface temperature up to 40 °C and maximum air temperatures by 0.5–2 °C (Rahman et al. 2020; McDonald et al. 2016). Evapotranspiration helps to reduce the energy available to warm ambient air by 1–8 °C as trees “breathe” out and transfer water vapor from surfaces into the atmosphere (Rahman et al. 2020, 2018; Park et al. 2021).

Reflective roofing, paving, and related products can increase the reflective capabilities of surfaces that often constitute substantial portions of densely-populated urban areas, thereby reducing urban temperatures (Kalkstein et al. 2020). The development and availability of these materials has increased in recent years. Through use of such products, urban areas can experience reductions of up to 2 °C during a dangerous heat event, which is sufficient to reduce heat-related mortality by 20% or more in some cities (Kalkstein et al. 2022).

This paper builds off previous research published by the Los Angeles Urban Cooling Collaborative (LAUCC) in 2022, which evaluated the effects of tree cover and cool surface scenarios on heat-related mortality in Los Angeles County (Kalkstein et al. 2022). LAUCC is a multi-disciplinary partnership of academic researchers and nonprofit organizations working with communities and government agencies to research and implement evidence-based strategies for mitigating urban heat and protecting heat-vulnerable communities from related health risks. In our prior study, we used historical meteorological and public health data to quantify how increases in tree cover and albedo of roofs and pavements in LA could reduce summer temperatures, decrease the number of oppressive air mass days — those leading to higher heat-health risks — and prevent heat-related deaths (Kalkstein et al. 2022).

For LA County as a whole, results of that study showed temperature reductions of up to 2.0 °C, leading to mortality reductions between 10 to 30%, depending on the tree cover + albedo scenario (Kalkstein et al. 2022). A more granular, district-level analysis used the conservative assumption that tree canopy/albedo increases would only occur in each district while the rest of the county’s land cover would remain unchanged. That analysis revealed that mortality reductions between 20 and 40% were common under various scenarios, with the largest numbers of lives saved in low-income communities of color.

We also found that climate change-induced warming could be delayed approximately 40 to 70 years under business-as-usual and moderate mitigation scenarios, respectively. That is, the warming caused at the global level could be counteracted at the local level by virtue of cooling modifications to the urban environment. Combined with other measures, increasing tree canopy and albedo clearly holds promise for reducing heat-health risk and saving dozens or hundreds of lives in the Los Angeles regions during the hottest years.

There is a considerable research gap between work on heat related *mortality* and *morbidity*, the latter of which is the focus of the present study. Working with morbidity is more challenging because data are more difficult to obtain and often lack standardization between geographic locations and medical facilities. As well, morbidity data are more nuanced. Data collected in emergency rooms or via emergency calls involve patients in various levels of infirmity, which makes data more opaque. Thus, there is often reticence to dive into morbidity data as compared to mortality data, contributing to the paucity of studies relating to heat and morbidity.

The goal of the present, follow-on study was to quantify the effect that similar scenarios could have on decreasing heat-induced morbidity (rather than mortality), as measured by emergency room (ER) visits. We tested the impacts of several scenarios for LA County and at a smaller geographic scale for a heat-vulnerable area in the northeast San Fernando Valley, a hot, inland area of LA.

## Materials and methods

### Overview

We first identified four historical summer heat waves between the years 2006 to 2010. Using a distributed lag non-linear model (DLNM), we evaluated the overall relationship between weather conditions (using the Excess Heat Factor, which combines the impact of temperature, humidity, and acclimatization) and morbidity within Los Angeles County (Nairn and Fawcett 2015). Los Angeles County as a whole was assessed, along with an analysis focused on a single heat-vulnerable neighborhood. The relationships that were derived were then used to estimate overall increases in heat morbidity during these four events. We created four alternate scenarios of changed urban forest cover and roof albedo, and then using a regional atmospheric model, simulated changes in atmospheric conditions for each of these scenarios. These conditions were then inputted back into the relationships derived previously, to estimate the possible impacts of a changed urban form on morbidity outcomes during heat events. The specific aspects of these methods are discussed below.

### Selection of heat waves

To select the heat waves, we used a “synoptic” climatological approach, which classifies days into one of several discrete air mass types that traverse a region and provide unique weather characteristics to that geographic area. In contrast to the separate analysis of temperature, humidity, and other meteorological variables, this approach offers a holistic evaluation that leads to effective pinpointing of deleterious conditions that lead to unusually high health impacts (Hondula et al. 2014). This is important because human health responds to a suite of variables that impact the individual simultaneously. Using the Spatial Synoptic Classification (Sheridan 2002), two particular air masses have been found in prior studies to be associated with statistically significant higher mortality rates, particularly during the summer months: dry tropical (DT), which produces oppressively hot and dry conditions, and moist tropical plus (MT +), excessively hot and humid (Sheridan and Kalkstein 2004; Lee and Sheridan 2018). These are the air mass types we focused on.

We used the same historical heat waves that we used for the study on mortality impacts discussed in Kalkstein et al. (2022). Note that the heat waves occur at different times during the summer season. While these are historic heat waves, they represent the types of heat waves that the LA region is likely to continue to experience, even if the frequency and duration are likely to change in a warming climate (California Energy Commission n.d.).

- July 22–26, 2006: hot and humid, dominated by MT + air mass days.
- June 19–23, 2008: a drier event with a mixture of MT and DT days.
- August 25–30, 2009: the least excessive heat wave of the four, with a mix of DT and TR (Transitional) days, to allow us to evaluate a more common situation that was not extreme.
- September 25–29, 2010: a very hot Santa Ana event with DT days.

### Study regions within Los Angeles County

We then tested the impacts of four different tree cover and albedo prescriptions for LA County as a whole, with a population of nearly 10 million and a land area of 4,057 square miles, as well as at a smaller geographic scale for a heat-vulnerable area centered around the northeast San Fernando Valley, located near the northern part of the City of Los Angeles (U.S. Census Bureau 2021). San Fernando has a large minority population, many of whom live in conditions that raise heat risk (i.e., older housing stock, poor insulation, or limited access to air conditioning). San Fernando’s harsh summer climate means its residents contend with greater heat exposure than residents in other parts of LA County with more benign climate zones, and some San Fernando neighborhoods experience twice as many ER visits on hot days compared to the county’s rate of 1.5 excess visits per 10,000 people (University of California Los Angeles 2022).

**Fig. 1:**
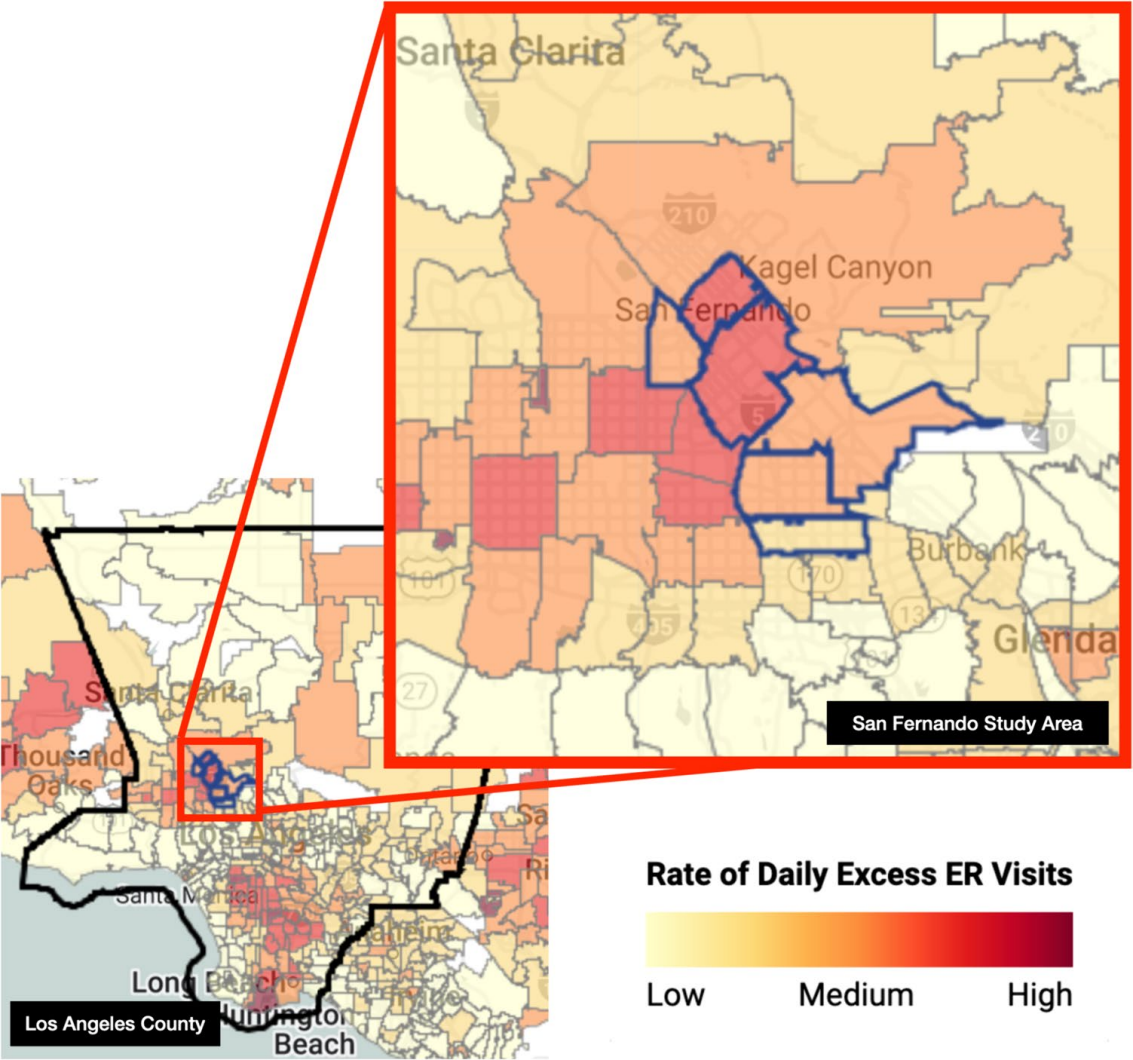
Map showing the rate of daily excess ER visits at the ZIP code level in Los Angeles County and the smaller study area (University of California 2022)

The public health data procured for this project are available at the ZIP code level of resolution, and modeling of San Fernando was conducted on a contiguous area demarcated at this geographic unit (Fig. 1). The total area is 93 sq. km. (36 sq. mi.), with a population of approximately 300,000 people living in mostly working-class communities that are more than 70% Latino/a (U.S. Census Bureau 2021). Two-thirds of the census tracts are rated at the 75th percentile or above for pollution burden and environmental vulnerability according to the CalEnviroScreen index, which is used as an indicator of how disadvantaged a population is (Office of Environmental Health Hazard Assessment 2021). This was one of the 11 districts modeled in the mortality study published in 2022, and is referred to as “District 9” in Kalkstein, et al. (2022). In that initial study, we segmented LA County in order to determine region-by-region variations in heat/health sensitivity as well as the effectiveness of various tree cover and albedo interventions. The districts were designed to be as homogeneous as possible in terms of demographics, socio-economic status, meteorology, and climate (Kalkstein et al. 2022; de Guzman et al. 2020).

### Morbidity data

ER visits are multiple times greater than the number of hospitalizations at a ZIP code scale. Using ER visits provides the statistical power to detect differences where hospitalization would not. ER visits are one common metric of health impact but one which has limitations including that it includes less severe levels of health impact than does hospitalization.

We examined four different subsets of emergency room visits with daily data acquired from the California Department of Health Care Access and Information for the period 2005–2018. All internal causes include non-external causes, such as those caused by cardiac, respiratory, renal, or diabetes-related issues. Heat-related causes include those which received a diagnosis of one or more of relevant International Classification of Diseases (ICD) codes. The following ninth-revision codes (ICD-9) were used for the years 2005–2015: E900.0 (excessive natural heat) and E992 (effects of heat and light). A new classification was adopted in 2016, and the following ICD-10 codes were used for the years 2016–2018: X30 (exposure to excessive natural heat) and T67 (effects of heat and light). The four subsets using these categories include:

1. **ER visits due to all internal causes in LA County**. While this clearly includes many visits unrelated to the weather, it has been widely recognized that hospitalizations and mortality increase during hot weather for more than just what are categorized as “directly heat-related” health impacts (Hajat et al. 2006).
2. **ER visits that are directly heat-related in LA County**. While these totals are much smaller than those from all internal causes, exploring direct heat-related morbidity outcomes provides additional insight into the most directly observable impacts of heat.
3. **ER visits due to all internal causes in San Fernando, a smaller heat-vulnerable portion of LA County.**
4. **ER visits that are directly heat-related in San Fernando**.

### Excess heat factor calculation

Heat exposure in this work is defined using a modified version of the Excess Heat Factor (EHF) developed by Nairn and Fawcett (2015), modified in Sheridan et al. (2019) by changing temperature to apparent temperature. The EHF considers the mean apparent temperature of the most recent three days against that of the prior 30 days. Thus, hot periods which follow relatively cool weather produce the highest EHF, and a number of studies have shown EHF to be a strong predictor of heat-related mortality (Langlois et al. 2013; Oliveira et al. 2022). Statistically, EHF is determined by calculating a product of the magnitude of the heat event, and an acclimatization term.

The magnitude of the heat event, expressed as excess heat (EH), is calculated as

1 $$EH={\text{max}}\left(0, \left({\sum }_{i=-2}^{0}{AT}_{i}\right)/3-AT95\right)$$

where ATi is the apparent temperature on day i, averaged over a three-day period, and AT95 is the overall 95th percentile of apparent temperature (based on the 1981–2010 normal period) for a given location.

The acclimatization term is defined as:

2 $$EHaccl=\left({\sum }_{i=-2}^{0}{AT}_{i}\right)/3-\left({\sum }_{i=-32}^{-3}{AT}_{i}\right)/30$$

The difference between the three-day mean apparent temperature and the 30 days prior. The Excessive Heat Factor is the product of these two terms,

3 $$EHF=\mathrm{max }\left(0, EH\right)\times \mathrm{max }\left(1, {EH}_{accl}\right)$$

in units of K^2^.

Per Nairn and Fawcett (2015), an extreme heat event (EHE) is defined as an instance when the EHF exceeds the 85th percentile of all positive EHF values for a given location over the climatological period. For the period of study, the greatest EHF observed is 84.8, and the threshold for an EHF is 25.5.

### Calculating relative risks of morbidity based on excess heat factor

We used historical data and calculated relative risk, or the likelihood of going to ER on a given heat wave day compared to if the heat wave had not occurred. Hence, a factor of 2 or a 200% increase represents double the likelihood of ER visits. We also calculated the data as visits per 100,000 people. This enables comparison of heat vulnerability between regions.

We calculated the relative risks (RR) of morbidity for each of the four data sets: countywide ER visits, countywide heat-related ER visits, local ER visits, and local heat-related ER visits. For each of these, we calculated relative risks using a distributed-lag non-linear model (DLNM) that assesses the cumulative impact of weather on morbidity, using the DLNM package in R. The model is:

$$\mathrm{Log }\left({\text{Morbidity}}\right)={\text{intercept}}+{\text{EHF}}+{\text{ns}} \left({\text{time}}\right)$$

where:

- morbidity is the daily total of ER visits from the relevant data set;
- ns (time) is a natural spline fit to the full 14-year period with 9 degrees of freedom per year to account for long-term changes in baseline morbidity as well as seasonal variations; and
- EHF is the excess heat factor defined above.

Each model examines the cumulative impact of heat over a 3-day period, allowing a comprehensive evaluation of any potential lags in ER visits due to heat.

### Alternate scenarios of tree cover and albedo

The next step in the project was to estimate how the various prescriptions in tree cover and albedo would impact local meteorology — the results of which are then used to determine impacts on health. We selected three distinct prescriptions plus a present-day baseline case (Table 1). The four scenarios vary considerably in tree cover and albedo of pavements, walls, and roofs. The tree cover scenarios were: Low (25% *relative* increase above baseline); Medium (50% *relative* increase above baseline); and High (40% total *absolute* tree cover, regardless of baseline, considered a maximum tree cover dictated by factors including LA’s climate, annual rainfall, and development patterns). Albedo values in Table 1 indicate the reflectance of each surface, with 0 being completely non-reflective and 1 being fully reflective.

**Table 1:** Tree cover and albedo scenarios. Tree cover percentages indicate the baseline proportion of land covered by tree canopy, followed by increases for each of the scenarios

Scenario	Tree Cover		Albedo			
	LA County	San Fernando	Flat Roof	Steep Roof	Pavement	Walls
Baseline	18%	20%	0.30	0.10	0.10	0.35
Low	22.5%	25%	0.45	0.20	0.20	0.45
Medium	27%	30%	0.63	0.30	0.30	0.50
High	40%	40%	0.75	0.35	0.35	0.60

To determine baseline cover values for trees, roofs, roads and other surfaces, we used the Los Angeles County Tree Canopy Advanced Viewer, a parcel-level LiDAR-based land cover assessment of LA County.^1^ To establish baseline values for roof and pavement albedo and determine increased scenarios, we consulted pertinent literature and resources, including the Lawrence Berkeley National Laboratory Hot Roofs, Cool Roofs mapping tool.^2^ Tree canopy and albedo scenarios were developed for both the countywide analysis and the district analysis. We determined a baseline number representing existing tree cover, roof albedo (two numbers — one for flat and another for steep roofs, as flat roofs tend to have higher albedo), pavement albedo, and wall albedo.

### Simulations of atmospheric conditions under alternate scenarios

To explore the effects of increasing tree cover or albedo at the district scale, we used a leading regional scale atmospheric model called the Weather Research and Forecasting (WRF) model, version 3.8.1 (Chen et al. 2011). Albedo modifications are implemented directly in the model input file by modifying albedo of roof and paving surfaces. However, increase in tree cover is modeled in a mosaic tile approach in which the tree cover of the non-urban land cover tiles was increased. This represents a limitation whereby the albedo modifications in urban tiles and the vegetation augmentation in non-urban tiles do not directly interact. The WRF model is routinely used to simulate urban climates, considering the effects of individual buildings and the various processes occurring within an urban area. High-resolution urban modeling with WRF is often limited to 1 km grid cells to ensure appropriate representation of parameterized model physics (Falasca and Curci 2018). However, if implemented carefully, and with thorough evaluation, models with nested grids of 500 m resolution can produce useful results (Nadimpalli et al. 2022; Shen et al. 2019). For this study, we simulated LA County using three nested domains. The interior domain covered all of LA County with 156 by 156 grid cells of 500 m per side. Atmospheric modeling details and model performance verification are provided in Kalkstein et al. (2022). The model used the single-layer urban canopy parameterization of Kusaka et al. (2001). Simulations were built using atmospheric initial and boundary conditions derived from the National Centers for Environmental Prediction’s North American Reanalysis (NARR) 3-hourly atmospheric data. Basic model parameterizations drew from multiple sources, including: the Rapid Radiative Transfer Model (RRTM) with the Dudhia shortwave radiation scheme; Monin–Obukhov (Janjic Eta) similarity scheme for the surface layer; the Mellor-Yamada-Janjic TKE scheme for boundary layer physics; and the Noah Land Surface Model. All simulations included a 7-day spin up period. Model performance was assessed by comparing the model output to hourly temperature recorded at several National Weather Service airport stations within the domain. Model RMSE values were generally 2–3 °C).

## Results

### How heat impacts ER visits

**Fig. 2:**
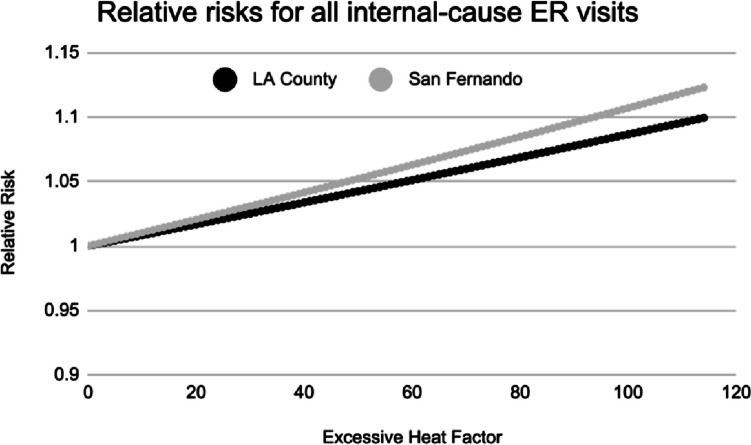
The relationship between excess heat factor (EHF) and relative risk (RR) of emergency room visits due to all internal causes for Los Angeles County as a whole (black) and San Fernando (gray)

As shown in Figs. 1 and 2, there is an increase in ER visits as the excess heat factor increases, across all four data sets analyzed — indicating that historical public health data records corroborate that heat waves send more LA residents to the ER. In Fig. 2, the relative risk of an ER visit is linearly related to excess heat, when considering ***all internal-cause ER visits***. Using Nairn-Fawcett Criteria (Nairn and Fawcett 2015) a heat event day would be considered to be one with an EHF between 40 and 80, and an extreme heat event day as one with an EHF of 81 or higher. For LA County as a whole, these thresholds are associated with a relative risk of 1.034 and 1.069 respectively, translating to 3.4% and 6.9% expected increases in ER visit totals across all internal causes. For San Fernando, the relative risks are slightly higher, at 1.042 and 1.086, respectively, suggesting that residents of the district are slightly more susceptible to the impacts of heat than residents in the rest of the County as a whole. In San Fernando, we thus expect to see approximately 4.2 to 8.6% more visits to the ER during heat waves.

**Fig. 3:**
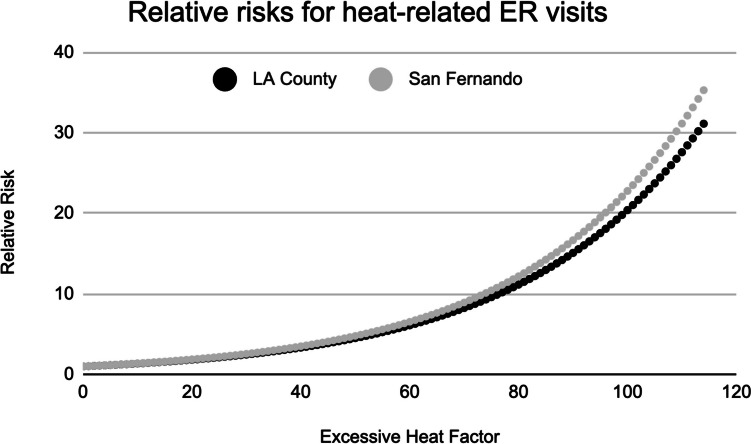
The relationship between excess heat factor (EHF) and relative risk (RR) of heat-related emergency room visits for Los Angeles County as a whole (black) and San Fernando (gray)

In Fig. 3, the corresponding relationships between EHF and relative risk of ***heat-related ER visits*** are shown. As heat-related ER visits are uncommon outside of hot weather, the relative risk values are much greater, with a curvilinear relationship that suggests extreme impacts on the hottest days. For LA County as a whole, a heat event is associated with a relative risk of 3.44 (which is 2.44 above a factor of 1, indicating a 244% increase in expected ER visits). As an EHF rises — indicating a more extreme heat wave — so too does relative risk. An extreme heat event is thus associated with a relative risk of 11.51 (1051% increase). As with overall ER visits, San Fernando is slightly more sensitive, with relative risks of 3.49 and 12.58.

### Impacts of cooling scenarios on heat stress conditions

Though the four heat events are all simulated for the same length of time (focused on the five hottest days of a given period), it is clear from Tables 2 and 3 that there are differences in magnitude of the heat events, with the very hot and humid 2006 event (MT +) by far the most extreme, followed by the hot, dry Santa Ana event of 2010 (a DT event). In comparing the response in terms of mean apparent temperature to that of EHF, it is clear that even modest reductions in temperature can reduce EHF substantially, due to its cumulative nature. The potential changes in meteorological conditions, as expected, are more pronounced under the more drastic scenario than the more moderate scenarios, with the magnitude of change varying across events. The 2009 and 2010 heat events, the two drier heat waves, show a greater reduction in mean apparent temperature resulting from the interventions than the more humid events of 2006 and 2008.

**Table 2:**
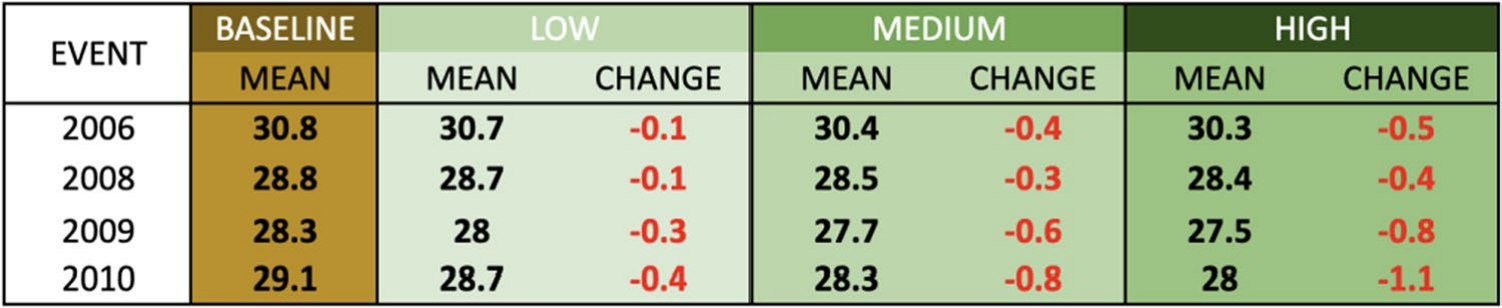
Mean apparent temperature (°C) for each of the four heat waves in the study

**Table 3:**
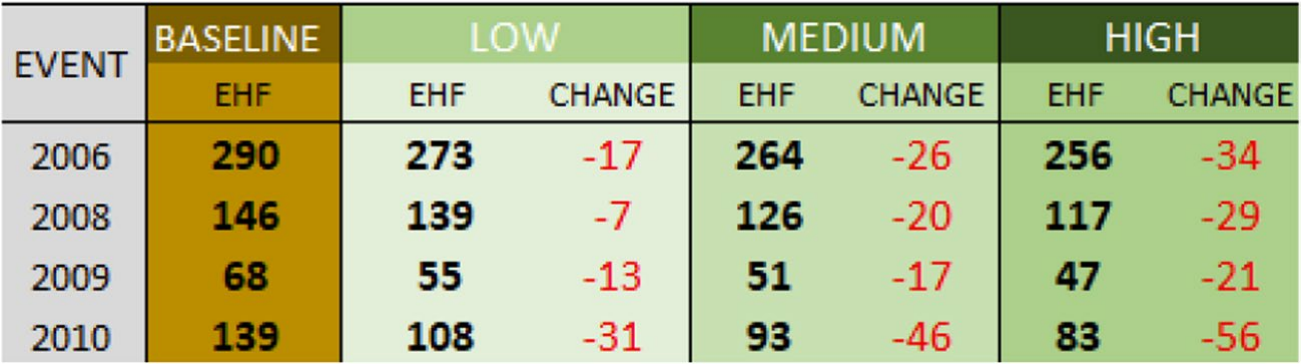
Modeled Excess Heat Factor for each of the four heat waves in the study

### Impacts of cooling scenarios on ER visits due to all internal causes

The differences in the magnitude of the heat events translates directly into differences in health impacts. The 2006 event as modeled shows an additional 245 ER visits due to all internal causes in LA County (Table 4), and 10.4 additional visits just in San Fernando (Table 5). The rate of increase in ER visits is higher across San Fernando (3.41 per 100,000 people) than LA County (2.49 per 100,000) as a whole, as the district itself has a higher level of sensitivity as shown in Figs. 1 and 2. The other three events show a similar relationship but with smaller impacts.

**Table 4:**
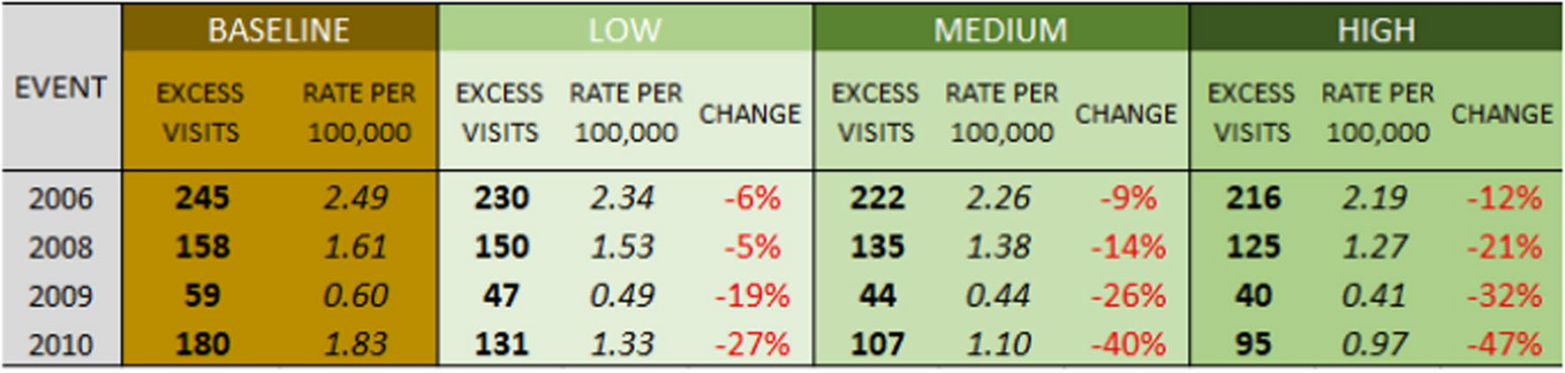
Modeled excess ER visits due to all internal causes, Los Angeles County

**Table 5:**
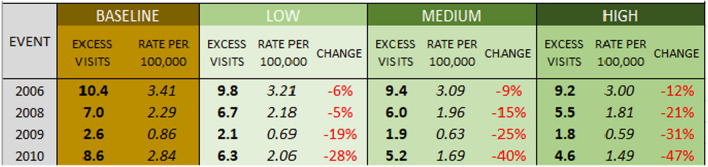
Modeled excess ER visits due to all internal causes, San Fernando

The three scenarios are all associated with a reduced impact on human health as measured by ER visits. Decreases for the humid 2010 event range from 28 to 47%, with the high scenario suggesting a decrease in 85 ER visits across Los Angeles County under the tree cover and albedo modifications. In the hottest event of 2006, though the improvement is lower due to modification leading to a less intense modeled reduction in air temperature, there is still a 12% decrease in ER visits projected under the High scenario, with a decrease of 29 ER visits across the county, from 245 to 216 ER visits.

Overall totals of *heat-related* ER visits are projected lower than all internal-cause visits, ranging across the four heat events from 15 to 145 visits in LA County (Table 6), and 1 to 6 visits in San Fernando (Table 7). Though the totals are lower, once again standardized rates of heat vulnerability per 100,000 people are higher across San Fernando than the county as a whole.

**Table 6:**
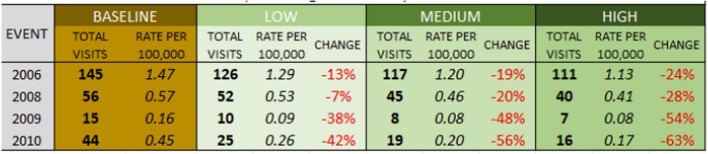
Modeled heat-related ER visits, Los Angeles County

**Table 7:**
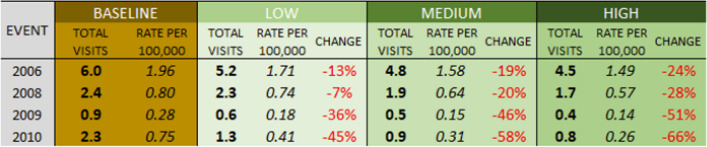
Modeled heat-related ER visits, San Fernando

The percentage decreases in heat-related ER visits under the scenarios are greater than the decreases in all-cause ER visits, given the direct connection between heat and the reason for the visit. Under the low scenario, heat-related ER visits are projected to drop 7% to 45% based on the event, and to drop between 24 and 66% under a more aggressive land cover scenario.

## Discussion

Together with the mortality study results presented in Kalkstein et al. (2022), the morbidity analyses conducted in this follow-on study demonstrate that by relying on a suite of interventions available today, Los Angeles has the capacity to cool its neighborhoods to a magnitude that would improve local-level meteorological conditions during extreme heat events. Though the meteorological outcomes of added tree cover and albedo vary based on heat wave characteristics — including seasonal timing, duration, intensity, and humidity — the cooling produced can lead to substantial decreases in both morbidity and mortality during heat events.

The previous study on mortality provided estimates on how many lives could be spared, demonstrating that upwards of 25% of lives lost could be spared during extreme heat waves if decisive action were taken to increase tree cover and the albedo of surfaces in the built environment. Air temperatures during the emblematic heat events we modeled decreased by 2–3 °C during the hottest periods of day as well as overnight, which is of particular importance for public health protection. The magnitude of change varies from event to event, with the greatest relative reduction in heat event impacts observed under the hot, dry conditions of 2010. Still, under all of these scenarios, heat events would still occur, but the magnitude of the heat events would be reduced to non-fatal thresholds for many vulnerable people.

**Fig. 4:**
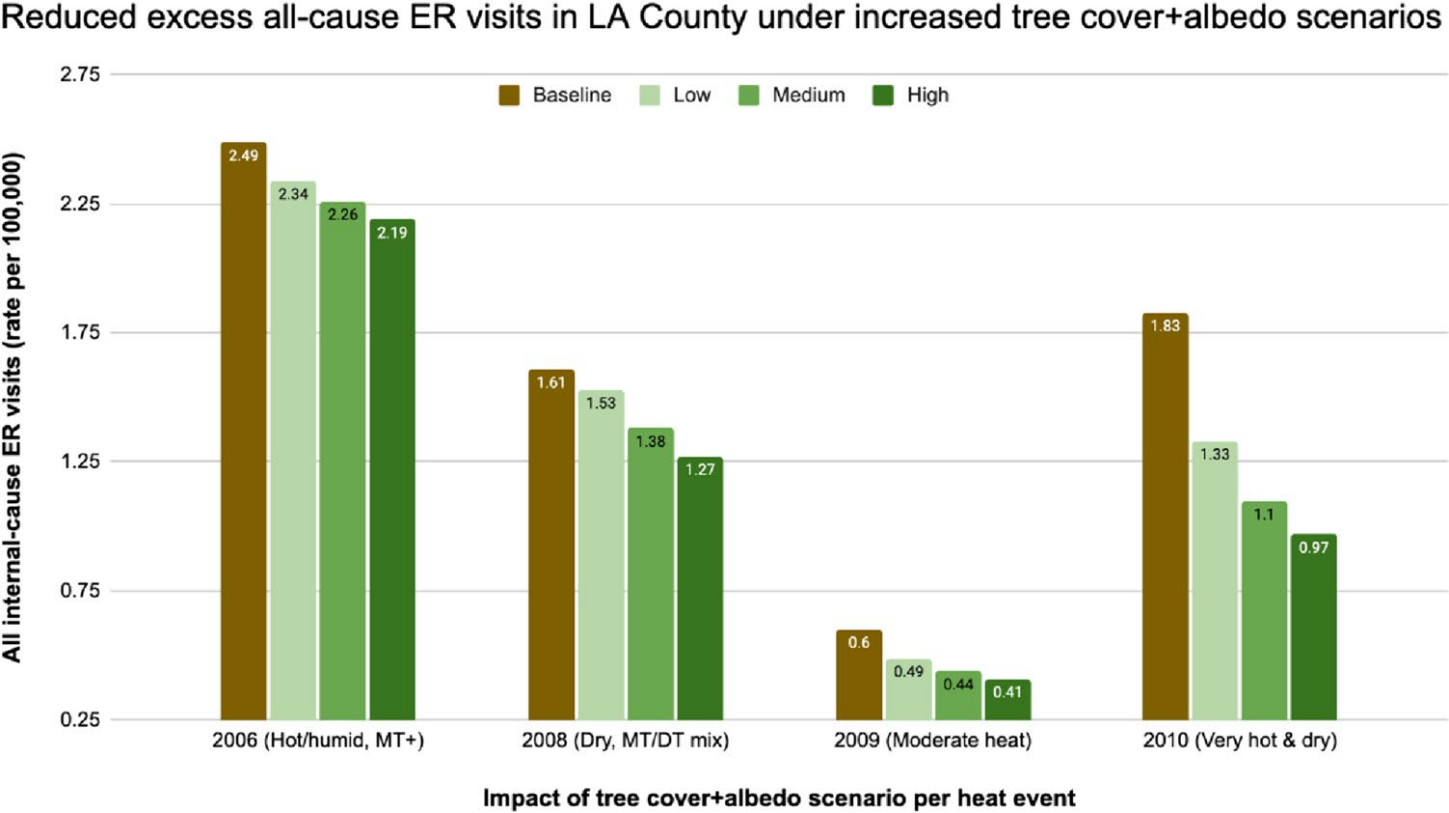
The impact of tree cover and albedo scenarios on reductions in the rate (per 100,000 people) of excess all-cause ER visits in LA County during the four heat events

In the present study, we evaluated the impacts that increased tree cover and albedo of roofs, pavements, and walls would have on morbidity, as measured by emergency room visits. Using the same four historical heat events we evaluated in the first study, we applied a modified version of the Excessive Heat Factor, a predictor of a heat event’s impact on public health outcomes. We found that, depending on the heat event, **ER visits due to all internal causes** in San Fernando and in LA County (Fig. 4) would be reduced:

- 5% to 27% under a low tree cover + albedo prescription
- 9% to 40% under a medium tree cover + albedo prescription
- 12% to 47% for a high tree cover + albedo prescription

We saw substantially higher reductions when focusing only on **heat-related ER visits**. For LA County and San Fernando, we saw reductions of:

- 7% to 45% under a low tree cover + albedo prescription
- 19% to 58% under a medium tree cover + albedo prescription
- 24% to 66% for a medium tree cover + albedo prescription

The findings of both studies indicate that we presently have the ability to address heat-related health impacts and reduce both heat-related illnesses and fatalities. As the planet gets hotter — and as cities warm at an accelerated pace — practical and actionable land cover solutions to a problem that disproportionately affects low-income communities are available and should be prioritized.

## Conclusion

The increasing use of reflective products for built environment surfaces and additional urban tree canopy cover can improve the quality of life in many densely-populated areas. This research demonstrates that during both moderate and extreme heat events, important temperature reductions and associated improvements in public health can be achieved by using strategies that are presently available. This is a key point: present strategies exist to greatly improve public health during heat events. Strategic changes in the land cover of urban areas can have significant and measurable positive benefits for public health, particularly for lower-income communities and people of color who suffer disproportionately from heat.

This research can be used by practitioners, policy makers, and advocates to encourage incentivization to motivate residents, private industry, and the governmental sector to move forward with programs leading to large-scale heat mitigation of urban areas. With the widespread availability of reflective products, and increased interest in and funding to support expansion of urban forests and other heat mitigation strategies, much can be achieved to make cities safer and more livable during excessive heat events.

## Data Availability

Data tables other than those presented in the manuscript will be made available upon request to the corresponding author.

## Footnotes

1 See https://tinyurl.com/treeviewer.

2 See https://albedomap.lbl.gov.
